# Validation of a Dutch version of the Tinnitus Functional Index in a tertiary referral tinnitus clinic

**DOI:** 10.1016/j.heliyon.2021.e07733

**Published:** 2021-08-10

**Authors:** Jose L. Santacruz, Rosemarie Arnold, Jolanda Tuinstra, Roy E. Stewart, Pim van Dijk

**Affiliations:** a Department of Otorhinolaryngology/Head and Neck Surgery, University of Groningen, University Medical Center Groningen, Groningen, the Netherlands; b Graduate School of Medical Sciences (Research School of Behavioral and Cognitive Neurosciences), University of Groningen, Groningen, the Netherlands; c University of Groningen, University Medical Center Groningen, Dept. of Health Sciences, the Netherlands; d University of Applied Sciences NHL Stenden, Dept. Health and Social Studies, Leeuwarden, the Netherlands

**Keywords:** Tinnitus, Questionnaire, Validation, Translation, Psychometric, Confirmatory factor analysis, Responsiveness, Treatment

## Abstract

**Introduction:**

Tinnitus is a condition with a subjective nature that requires self-report questionnaires for its assessment. Aspects such as quality of life, sleep or intrusiveness have been addressed by multiple tinnitus questionnaires, but the high responsiveness to treatment effects of the Tinnitus Functional Index (TFI) makes this questionnaire part of the standard practice in tinnitus screening. To date, the TFI has been translated to more than 20 languages and used in more than 22 countries. In this study, the TFI was translated to Dutch and validated through a clinical population in the Netherlands.

**Methods:**

After a back-translation procedure, the Dutch TFI was filled-out by 377 patients in the tinnitus outpatient clinic at the Ear, Nose and Throat (ENT) department of the University Medical Center Groningen, in the Netherlands. Reliability and construct validity of the questionnaire were assessed by correlations with one other tinnitus questionnaire (Tinnitus Handicap Inventory, THI) and with three psychological functioning questionnaires (Rand-36, Cantril's ladder and the Hospital Anxiety and Depression Scale (HADS)). The eight-factor structure of the Dutch TFI was tested by means of exploratory factor analysis using three different models (ICM-CFA, ESEM and ESEM-CFA).

**Results:**

The Dutch TFI showed a high internal consistency (α = 0.95), and construct validity was proven by moderate-to high-convergent correlations with the THI (r = 0.47–0.79) and by moderate convergent (r = 0.55–0.67) and good-to moderate-divergent (r = 0.12–0.47) correlations with the psychological functioning questionnaires. The eight-factor structure of the TFI was confirmed for the Dutch version by the three models.

**Conclusion:**

The Dutch version of the TFI is a reliable instrument for screening tinnitus impact in a clinical population, and its psychometric properties are comparable to the original TFI and other validated tinnitus questionnaires.

## 1 Introduction

Tinnitus (“ringing in the ears”) is usually defined as the perception of a sound for which no external sound source exists. Most people experience episodes of tinnitus at times (ringing, buzzing or other sounds), either spontaneously or after being exposed to loud noise. In most cases, these sounds diminish or disappear after a certain period of time, from a few minutes to several days. If this perception persists for a period of 6 months or longer, the problem is considered chronic tinnitus (Mazurek et al., 2010).

Tinnitus is a common complaint, but its mechanisms are still poorly understood. Although different theories have been proposed, consensus has arisen with respect to a “central model” for the etiology of tinnitus, which is built on the assumption that tinnitus is the result of a change in spontaneous neural activity in the central auditory system (Eggermont and Roberts, 2004; Norena, 2011). Most cases of tinnitus are associated with some degree of hearing loss (Shargorodsky et al., 2010). Disentangling the two of them is still a challenge today since hearing loss and tinnitus are closely related (Ratnayake et al., 2009): proportions from 70 % to 80 % of substantial hearing loss among tinnitus patients have been reported (Jastreboff, 2011). The prevalence of tinnitus in the adult population has been estimated to fall in the range of 10 %–15 % (De Ridder et al., 2014). Although there is no clear consensus in the literature on the association between sex and tinnitus (Gallus et al., 2015; Biswas and Hall, 2020), several studies have shown an increase in tinnitus prevalence and reported severity as a function of age (McCormack et al., 2014; Gallus et al., 2015; Bhatt et al., 2016). Despite clinical experience shows some examples of tinnitus in children, there is still a lack of a robust research on this issue (Rosing et al., 2016; Smith et al., 2019).

Although the consequences of tinnitus are diverse, for most patients these symptoms affect their quality of life (QoL) to a certain degree (Zeman et al., 2014). When patients severely suffer from tinnitus, several aspects of their daily functioning are also affected (Andersson and Westin, 2008). Consequences often reported by patients are sleep disturbance (Schecklmann et al., 2015), fatigue (Burke and Naylor, 2020), difficulties with hearing and with concentration (Mohamad et al., 2016), and a higher sensitivity to everyday sounds (hyperacusis (Schecklmann et al., 2014)). Relationships between tinnitus and psychological distress have been reported in several studies, highlighting that substantial percentages of the tinnitus patients had symptoms of depression or anxiety (Holmes and Padgham, 2009; Durai and Searchfiled, 2016).

Since the consequences of tinnitus can be significant, research has aimed at finding effective treatments for tinnitus (Dobie, 1999; Savage and Waddell, 2014), such as pharmacological, electrophysiological or psychological approaches (Hall et al., 2016). Since a cure for tinnitus has not yet been found, the treatment of patients with tinnitus has shifted towards tinnitus management (Henry et al., 2005; Hoare et al., 2011). Tinnitus management aims at assisting patients in living with their condition as good as possible and to improve their quality of life. In order to assess the effect of tinnitus treatments on managing the complaints, there is a need for standardised outcome measures. Numerous self-report questionnaires have been developed to assess the impact of tinnitus on patients’ quality of life (Meikle et al., 2008; Kamalski et al., 2010; Hall et al., 2016), although these questionnaires were not specifically designed to study treatment outcomes (Kamalski et al., 2010). In order to study the effects of treatment options on the quality of life of the patients, it is necessary to use instruments that are responsive to treatment effects (Meikle et al., 2008). Therefore, Meikle et al. (2012) developed the Tinnitus Functional Index (TFI), to be able to assess both the impact of tinnitus and the treatment-related effects on the quality of life of the patients. In the developing process, an original prototype consisting of 175 items belonging to 9 different tinnitus questionnaires were evaluated by an expert panel and 13 different domains or subcategories were identified. After a refining process of clinical evaluations and restructurations, the final TFI resulted in 25 questions organized in 8 subscales of factors: intrusive, sense of control, cognitive, sleep, auditory, relaxation, quality of life and emotional.

The aim of the present study is to assess the psychometric properties of a Dutch version of the Tinnitus Functional Index and to test whether the same structure of 8 factors can be found, taking into consideration how these factors relate to each other. The original English version of the TFI has recently been validated within several cultures and for different languages (Oron et al., 2018; Kam et al., 2018; Peter et al., 2017; Hoff and Kahari, 2017; Wrzosek et al., 2016; Fackrell et al., 2016, 2018; Rabau et al., 2014; Suzuki et al., 2019; Müller et al., 2016). It is worth noting that the TFI version of Rabau et al. (2014) is written in Dutch language from Belgium (also known as Flemish Dutch), different from the one proposed in our study. Here, the performance of the Dutch version of the Tinnitus Functional Index was studied in a clinical setting, as part of the assessments in a tinnitus outpatient clinic at the ENT department of a university hospital in the Netherlands.

## 2 Materials and methods

### 2.1 Participants and procedure

As part of a standard diagnostic protocol, the data for this study were collected in a tertiary referral tinnitus clinic at the University Medical Center Groningen. All patients who visited this clinic filled in several questionnaires in order to gather information on their tinnitus characteristics as well as to screen for potential psychosocial problems. These data are used in the multidisciplinary assessment of the patients to determine the advice for further treatment. The Dutch version of the TFI was administered to a group of 377 consecutive tinnitus patients, who visited the specialised multidisciplinary outpatient clinic between September 2013 and September 2015.

Data were included in this study when patients were 18 years or older, and mastered the Dutch language sufficiently to fill in the questionnaires. Since the data were collected as part of the routine assessment in the tinnitus outpatient clinic and are anonymously reported in this paper, no informed consent was asked of the participants. The study met the criteria for an exemption from institutional review board approval (METc2013/400).

### 2.2 Measurements

#### 2.2.1 The Tinnitus Functional Index

The original TFI (Meikle et al., 2012) was translated by means of a back-translation procedure, following Guilliman et al. (1993) guidelines. First, the translation to Dutch was carried on by two independent translators with Dutch as native language. Our Dutch translation of the questionnaire was translated back into English by another translator with English as native language. Thus, the accuracy of the translation process could be checked. None of the translators involved in the process were medically skilled. The comparison of the original TFI with the translated version was carried out by bilingual experts in the field, and it did not reveal differences in the meaning of the individual items.

**Figure 1:**
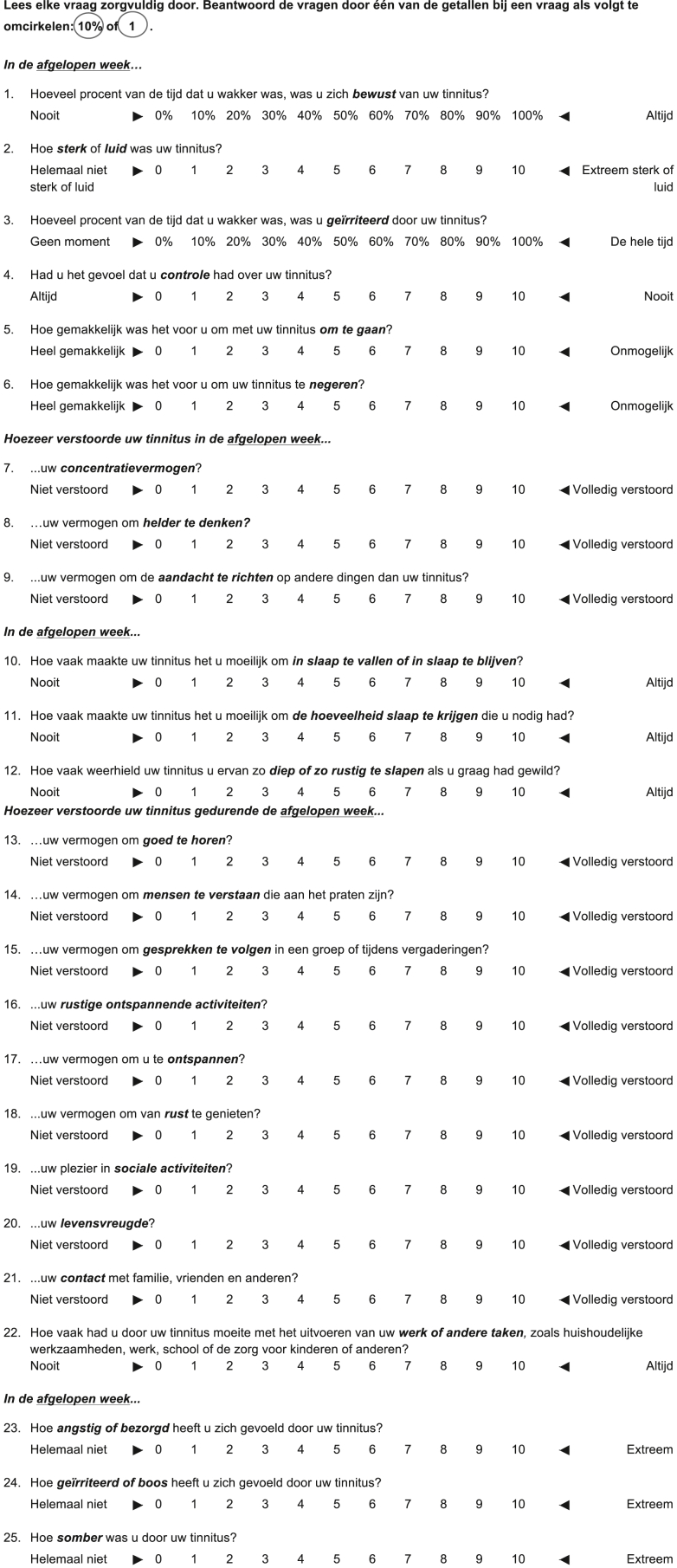
Dutch version of the Tinnitus Functional Index.

The TFI consists of 25 items, which are divided into 8 subscales: intrusive (3 items), sense of control (3 items), cognitive (3 items), sleep (3 items), auditory (3 items), relaxation (3 items), quality of life (4 items), and emotional (3 items). All items are scored on a 10-point rating scale, with “0” and “10” indicating the lowest and highest impact on functioning, respectively. Items 1 and 3 are scored as percentages and have to be re-coded into a 10-point scale. Each subscale is scored individually: scores on the separate items are added up, divided by the number of items in the scale, and multiplied by 10. For the total TFI score, all items are added up, divided by 25 (the total number of items) and multiplied by 10. Figure 1 shows the Dutch version of the TFI. Total scores between 0-17 are interpreted as “not a problem”, total scores between 18-31 as “small problem”, total scores between 32-53 as “moderate problem”, total scores between 54-72 as “big problem”, and total scores between 73-100 as “very big problem”.

#### 2.2.2 Tinnitus Handicap Inventory

In the present study, scores on the TFI were compared to scores on the Dutch version of the Tinnitus Handicap Inventory (THI; Newman et al., 1996), a validated (Newman et al., 1998; Brussee, 2003) and widely used questionnaire developed to assess the severity of patients’ tinnitus handicap. The THI consists of 25 items, scored on a 3-point self-rating scale (0 = “no”, 2 = “sometimes”, and 4 = “yes”). In addition to the total score, three different subscales are scored as well: functional (11 items), emotional (9 items), and catastrophic (5 items). Higher scores indicate a higher tinnitus impact.

#### 2.2.3 Psychological functioning

*Mental health or psychological functioning* was measured by the Mental Health subscale of the Rand-36 Health Survey (Ware, 1992; Vander Zee et al., 1996). This subscale consists of 5 items, scored on a 6-point self-rating scale (0 = never to 6 = always) and assesses mood, including symptoms of depression and tension. The total score on this subscale varies from 0 to 100, with higher scores indicating a better psychological functioning or mental health.

*Overall wellbeing* was measured on Cantril's ladder (Cantril, 1965), which is a scale ranging from 0 to 10. Patients answered the following question: ‘Here is a picture of a ladder. Suppose the top of the ladder represents the best possible life for you and the bottom represents the worst possible life for you. Where on the ladder do you feel you personally stand at the present time?’

*Symptoms of anxiety or depression* were assessed by the Hospital Anxiety and Depression Scale (Zigmond and Snaith, 1983; Spinhoven et al., 1997). The HADS is a 14-item self-report screening instrument, developed to identify possible cases with anxiety or depression. The instrument consists of two 7-item scales, one of them with items assessing symptoms of anxiety, and the other one with items assessing symptoms of depression. The subscales vary from 0 to 21, with higher scores indicating a higher amount of anxiety or depression. The authors of the original questionnaire identified scores from 0 to 7 as “non-cases”, scores from 8 through10 as “doubtful cases”, and scores higher than 11 as “cases” with anxiety or depression (Zigmond and Snaith, 1983).

### 2.3 Data analysis

All descriptive analyses, reliability analyses, and construct validity analyses were performed with IBM SPSS Statistics 23. The factor structure of the Dutch version of the TFI was tested with M-Plus version 8.

#### 2.3.1 Reliability and construct validity of the TFI

Reliability scores of the Dutch TFI were assessed by calculating the internal consistency coefficient Cronbach's alpha (α) for each subscale as well as for the total questionnaire (Cronbach, 1951). In general, Cronbach's alphas of ≥ .80 are considered good for diagnostic instruments, although Cronbach's alphas of > .90 are recommended in case of screening instruments (Nunnally, 1994).

Construct validity was evaluated by means of convergent and divergent correlations between TFI and measures of tinnitus handicap and psychological functioning. For it, Spearman's correlation coefficients between these measures were obtained. Correlation coefficients between .10 and .30 were considered small, correlations between .30 and .50 were considered moderate, and correlations higher than .50 were considered large (Cohen, 1988).

#### 2.3.2 Factor structure of the TFI

In the original study (Meikle et al., 2012), the eight-factor structure of the TFI was derived from a principal component analysis (PCA, aimed to reduce the dimensionality of data) as an independent cluster model (ICM). An ICM (Marsh et al., 2009) is a factor structure in which each of the 25 items is loaded on only one of the eight factors. Three models were tested and compared to confirm the factor structure of the TFI.

First, a confirmatory factor analysis (CFA) was performed to check whether the ICM eight-factor structure of the original study could be confirmed (model ICM-CFA). In an ICM model, items load at their respective factor with no cross-loads on the other latent factors. A critical comment on the ICM model is that the zero factor loadings of items usually displays poor fit and leads to distorted factors with overestimated factor correlations (Marsh et al., 2009).

Second, in order to investigate whether cross-loading could be found in the ICM-CFA, an exploratory structural equation model (ESEM) (Asparouhov and Muthén, 2009) of eight factors was performed as an exploratory factor analysis (EFA).

And third, based on the ESEM model, we investigated whether an ESEM-CFA model could be obtained. An ESEM-CFA model means that non-significant loadings that are larger than zero of the ESEM solution then become zero loadings. This involves obtaining a model with cross-loadings, but the cross-loadings were retrieved from an ESEM model. Since the data were comprised of continuous variables, parameter estimation of the ESEM model was estimated by maximum likelihood (ML) with oblique factor rotation Geomin. A Geomin criterion of 0.01 with 30 random starts was used.

Finally, a goodness of fit test (GOF) (Schreiber et al., 2006) was used to compare the three models (ICM-CFA, ESEM and ESEM-CFA).

## 3 Results

### 3.1 Participants

Table 1 shows the demographic characteristics of the patients that were included in this study. In total, 377 patients participated in the study. More men (60.7 %) than women (39.3 %) were included, with a mean age of 54.8 years (range 19–88 years). Tinnitus duration was on average 7.1 years. The number of patients with an acute or gradual onset of tinnitus was almost equally divided. Most of the patients in this study experienced a continuous tinnitus (89.6 %), whereas a smaller amount of the patients experienced tinnitus at intervals (10.4 %). The majority of the patients reported hearing loss (68.7 %). The demographic data described a wide range of characteristics in our clinical population.

**Table 1:** Demographic data and tinnitus characteristics of the subjects.

Demographic characteristics	N = 377 (%)
Gender	
Male	229 (60.7)
Female	148 (39.3)
Age (years)	
Mean	54.8
SD	13.6
Range	19–88
Marital status	
With partner	301 (80.3)
Without partner	74 (19.7)
Missing	2
Educational level	
Low	87 (24.0)
Middle	166 (45.7)
High	110 (30.3)
Missing	14
Tinnitus duration (years)	
Mean	7.1
SD	8.1
Range	0–47
Onset of tinnitus	
Acute	174 (47.7)
Gradual	191 (52.3)
Missing	12
Presence of tinnitus	
Continuous	329 (89.6)
With intervals	38 (10.4)
Missing	10
Perceived hearing loss	
No	118 (31.3)
Yes	259 (68.7)

### 3.2 Instruments

Table 2 gives an overview of all instruments used in the present study. The average TFI score fell into the ‘moderate problem’ category with a value of 48 ± 20.4, characteristic of a common tinnitus population as previous studies reported (Fackrell et al., 2018; Wrzosek et al., 2016; Peter et al., 2017; Jacquemin et al., 2019). In line with it, the THI presented also a ‘moderate handicap’ on average with a score of 44 ± 22.3. Psychological functioning tests such as Rand-36, Cantril's ladder and HADS presented relatively normal average values as well.

**Table 2:** Questionnaires and subscales used. The maximum score for TFI, THI and RAND-36 is 100. The maximum scores for Cantril's ladder and HADS is 10 and 21, respectively.

	N	Items	Possible Score Range	Observed Score Range	Mean	SD
Tinnitus Functional Index						
Intrusive	356	3	0–100	0–100	61.61	21.98
Sense of Control	359	3	0–100	3.33–100	65.04	20.44
Cognitive	362	3	0–100	0–100	43.43	25.38
Sleep	369	3	0–100	0–100	47.27	34.29
Auditory	361	3	0–100	0–100	43.52	30.50
Relaxation	366	3	0–100	0–100	47.48	27.96
Quality of Life	360	4	0–100	0–100	37.26	26.74
Emotional	363	3	0–100	0–100	39.61	27.66
Total	371	25	0–100	3.20–100	47.93	20.41
Tinnitus Handicap Inventory						
Functional	368	11	0–44	0–44	21.68	10.41
Emotional	368	9	0–36	0–36	14.08	9.01
Catastrophic	370	5	0–20	0–20	7.96	4.88
Total	374	25	0–100	0–98	43.84	22.33
RAND-36						
Mental health	372	5	0–100	0–100	63.89	19.27
Cantril's ladder	363	1	0–10	0–10	6.31	1.85
Hospital Anxiety & Depression Scale						
Anxiety	368	7	0–21	0–21	6.96	4.18
Depression	369	7	0–21	0–21	5.82	4.54

### 3.3 Reliability

Table 3 summarizes the internal consistency scores of the subscales of the Dutch version of the TFI. Most of the subscales of the TFI, as well as the total scale, showed good internal consistency scores (Cronbach's alphas ranged from 0.82‒0.96). Subscale “sense of control” showed a satisfactory internal consistency with a Cronbach's alpha of .72.

**Table 3:** Internal consistency scores of the Dutch version of the TFI.

	N	Items	Cronbach's Alpha
Tinnitus Functional Index			
Intrusive	356	3	.82
Sense of Control	359	3	.72
Cognitive	362	3	.92
Sleep	369	3	.96
Auditory	361	3	.95
Relaxation	366	3	.94
Quality of Life	360	4	.89
Emotional	363	3	.90
Total	309	25	.95

The internal consistency scores of the Dutch TFI were comparable to the scores of the original English version of the TFI, with only a lower internal consistency score for subscale “sense of control” of the Dutch version of the TFI (Meikle et al., 2012). The obtained values of internal consistency highlighted the reliability of each subscale.

### 3.4 Construct validity

Table 4 shows the expected convergent and divergent correlations between TFI subscales, THI subscales, and measures of psychological functioning. Convergent correlations were expected between the TFI subscales and the corresponding subscales of the THI. Also, subscales Quality of Life and Emotional were expected to be related to measures of psychological functioning. Divergent correlations were expected between TFI subscales Cognitive, Sleep and Auditory and measures of psychological functioning. These assumptions were made by the authors and based on their own clinical experience. In the case of the expected correlations between TFI and THI, both questionnaires contain similar questions.

**Table 4:** Expected convergent and divergent correlations between TFI subscales, THI subscales, and measures of psychological functioning.

	THI Functional	THI Emotional	THI Catastrophic	RAND-36 Mental Health	Cantril's Ladder	HADS Anxiety	HADS Depression
TFI							
Intrusive		+					
Sense of Control			+				
Cognitive	+			0	0	0	0
Sleep	+			0	0	0	0
Auditory	+			0	0	0	0
Relaxation	+					+	
Quality of Life	+	+		-	-		+
Emotional		+		-	-	+	+

+ = expected positive correlation. - = expected negative correlation. 0 = no association expected.

Table 5 displays the actual convergent and divergent correlations that were found in the study population. With respect to convergent validity, all TFI subscales showed significant moderate-to strong-correlations (range 0.47–0.79) with the corresponding subscales of the THI and measures of psychological functioning. Subscales Intrusive and Auditory correlated less strongly with THI subscales Emotional and Functional, respectively (r = 0.47). All expectations regarding the direction of the convergent correlations were confirmed by the results.

**Table 5:** Convergent and divergent Spearman correlations obtained between TFI subscales, THI subscales, and measures of psychological functioning.

	THI Functional	THI Emotional	THI Catastrophic	RAND-36 Mental Health	Cantril's Ladder	HADS Anxiety	HADS Depression
TFI							
Intrusive		**.47∗∗**					
Sense of Control			**.54∗∗**				
Cognitive	**.76∗∗**			*-.46∗∗*	*-.50∗∗*	*.47∗∗*	*.56∗∗*
Sleep	**.60∗∗**			*-.37∗∗*	*-.33∗∗*	*.39∗∗*	*.44∗∗*
Auditory	**.47∗∗**			*-.12∗*	*-.12∗*	*.19∗∗*	*.26∗∗*
Relaxation	**.64∗∗**					**.53∗∗**	
Quality of Life	**.79∗∗**	**.68∗∗**		**-.55∗∗**	**-.56∗∗**		**.67∗∗**
Emotional		**.78∗∗**		**-.66∗∗**	**-.57∗∗**	**.60∗∗**	**.64∗∗**

∗ = p < .05. ∗∗ = p < .01.

Bold values indicate the expected convergent correlations; Italic values indicate expected divergent correlations.

With respect to divergent validity, significant, but small-to moderate-correlations were found for TFI subscales Cognitive, Sleep, and Auditory with measures of psychological functioning. Almost all of the correlation coefficients were smaller than 0.50 (range 0.12–0.47), which is indicative of a satisfactory divergent validity. Subscale Cognitive correlated strongly with overall wellbeing as measured by Cantril's ladder (r = 0.50), which indicates that some association exists between these constructs. All expectations with respect to the direction of the divergent correlations were confirmed by the results.

Overall, the construct validity showed smaller divergent correlations compared to convergent correlations for the subscales of the TFI. These correlations indicated a strong construct validity of the questionnaire for almost all subscales, which might infer that these factors are adequate for assessing the aspects of tinnitus that they are intended to measure.

### 3.5 Confirmation of the 8-factor structure of the TFI

The 8-factor structure was tested by three different models (ICM-CFA, ESEM, and ESEM-CFA).

Tables 6A, 6B and 6C show the standardized factor loadings (β) for all 25 TFI items and the 8 factors. The loadings of the ICM-CFA are shown in Table 6A, where only the items of each factor are considered and the empty cells represent zero loadings. All values indicate good associations with their designated factor since they are above the recommended cut-off ≥ 0.40 (Wülferth, 2013). Table 6B contains the loadings of the ESEM model. Values in bold correspond to the significant loadings (p ≤ 0.05), which occurs for items that are either associated with their factor or not. For this model, several items showed significant cross-loadings with other factors (i.e., item 20 and factor Emotional). However, none of these cross-loadings scored above the cut-off value of 0.40. The loadings of the model ESEM-CFA are shown in Table 6C, which includes only the significant loadings obtained in the ESEM-CFA model, zero loadings appear blank. As in the previous model, none of the cross-loadings scored above 0.40.

**Table 6A:** Standardized loadings (β) of ICM-CFA model: Eight factors based on 25 items of the TFI. All values are above the recommended cut-off ≥ 0.40.

	Intrusiveness	Sense of Control	Cognitive	Sleep	Auditory	Relaxation	Quality of life	Emotional
TF1	0.778							
TF2	0.826							
TF3	0.770							
TF4		0.415						
TF5		0.849						
TF6		0.778						
TF7			0.903					
TF8			0.918					
TF9			0.839					
TF10				0.901				
TF11				0.985				
TF12				0.925				
TF13					0.918			
TF14					0.997			
TF15					0.897			
TF16						0.896		
TF17						0.957		
TF18						0.891		
TF19							0.868	
TF20							0.850	
TF21							0.812	
TF22							0.768	
TF23								0.781
TF24								0.875
TF25								0.949

**Table 6B:** Standardized loadings (β) of ESEM model: Eight ESEM factors based on 25 items of the TFI.

	Intrusiveness	Sense of Control	Cognitive	Sleep	Auditory	Relaxation	Quality of life	Emotional
TF1	**0.924**	-0.049	-0.068	-0.011	0.001	0.006	0.073	-0.011
TF2	**0.627**	0.134	0.051	0.010	**0.121**	0.065	-0.038	0.021
TF3	**0.448**	0.159	0.120	0.041	-0.034	-0.037	-0.015	0.268
TF4	-0.004	**0.473**	0.013	-0.090	0.089	0.080	0.044	-0.100
TF5	0.014	**0.593**	-0.003	0.098	0.010	-0.028	-0.030	0.380
TF6	0.078	**0.630**	0.042	0.011	-0.020	0.045	0.093	0.060
TF7	0.072	0.079	**0.736**	0.076	0.035	-0.004	0.064	-0.025
TF8	-0.002	-0.128	**0.992**	0.009	0.037	0.019	-0.014	0.013
TF9	-0.015	0.079	**0.607**	0.013	-0.041	0.085	**0.162**	0.074
TF10	0.012	0.044	0.069	**0.860**	-0.034	-0.015	-0.039	0.041
TF11	0.004	-0.022	-0.006	**0.962**	**0.035**	0.014	0.024	0.019
TF12	0.005	0.003	-0.006	**0.891**	0.016	0.063	0.041	-0.045
TF13	0.028	-0.020	0.008	-0.004	**0.893**	0.027	-0.013	0.046
TF14	-0.029	0.014	-0.037	0.017	**1.014**	0.017	-0.005	0.041
TF15	0.033	0.011	0.083	0.006	**0.815**	-0.029	**0.109**	-0.092
TF16	-0.003	0.018	0.070	0.019	**0.071**	**0.808**	0.010	-0.009
TF17	0.005	-0.039	0.055	0.024	0.004	**0.911**	0.010	0.019
TF18	0.037	0.052	-0.026	0.012	-0.020	**0.813**	0.018	0.063
TF19	0.054	0.019	0.004	0.004	0.035	0.029	**0.872**	-0.040
TF20	0.024	0.000	-0.019	0.041	**-0.086**	**0.142**	**0.520**	**0.353**
TF21	-0.028	-0.026	0.066	0.024	0.056	-0.120	**0.795**	0.101
TF22	0.001	0.079	0.295	-0.051	0.021	0.079	**0.452**	0.037
TF23	-0.039	0.052	-0.124	0.041	0.039	0.072	0.088	**0.714**
TF24	0.034	0.045	0.059	-0.068	0.053	0.023	-0.009	**0.818**
TF25	0.031	-0.096	0.077	-0.006	-0.007	0.010	0.067	**0.895**

Values in bold correspond to the significant loadings (p ≤ 0.05).

**Table 6C:** Standardized loadings (β) of ESEM-CFA model: Eight ESEM factors based on 25 items of the TFI. Only the significant loadings (p ≤ 0.05) of the ESEM-CFA model are shown, zero loadings appear blank.

	Intrusiveness	Sense of Control	Cognitive	Sleep	Auditory	Relaxation	Quality of life	Emotional
TF1	1.266	-0.330	-0.219					
TF2	0.806							
TF3	0.599							0.240
TF4		0.555						-0.153
TF5		0.646						0.260
TF6		0.819						
TF7			0.897					
TF8		-0.297	1.142					
TF9			0.685				0.204	
TF10				0.900				
TF11				0.985				
TF12				0.925				
TF13					0.915			
TF14			-0.075		1.040			
TF15					0.896			
TF16						0.895		
TF17						0.957		
TF18						0.890		
TF19							1.001	-0.135
TF20					-0.136	0.120	0.592	0.307
TF21						-0.193	0.979	
TF22			0.320				0.519	
TF23								0.779
TF24								0.872
TF25								0.955

Tables 7A, 7B and 7C contain the correlations between factors of the 3 models. Values presented in bold are below or above the recommended criteria (<0.30 to >0.85) (Hair et al., 2010). For all models, the Auditory factor showed the weakest correlations with the rest of the factors.

**Table 7A:** ICM-CFA model: Correlations between factors.

Factor	1	2	3	4	5	6	7	8
(1) Intrusiveness	1	0.766	0.624	0.495	0.388	0.590	0.588	0.595
(2) Sense of Control		1	0.646	0.505	**0.293**	0.632	0.614	0.676
(3) Cognitive			1	0.562	0.505	0.700	0.756	0.625
(4) Sleep				1	**0.239**	0.586	0.501	0.474
(5) Auditory					1	0.366	0.494	**0.269**
(6) Relaxation						1	0.749	0.696
(7) Quality of life							1	0.780
(8) Emotional								1

Values in bold are below or above the recommended criteria (<0.30 to >0.85). 1 = Intrusiveness; 2 = Sense of control; 3 = Cognition; 4 = Sleep; 5 = Auditory; 6 = Relaxation; 7 = Quality of life; 8 = Emotional.

**Table 7B:** ESEM model: Correlations between factors.

Factor	1	2	3	4	5	6	7	8
(1) Intrusiveness	1	0.547	0.456	0.393	0.312	0.446	0.381	0.433
(2) Sense of Control		1	0.488	0.342	**0.215**	0.444	0.320	0.412
(3) Cognitive			1	0.483	0.467	0.605	0.607	0.523
(4) Sleep				1	**0.177**	0.527	0.384	0.434
(5) Auditory					1	0.308	0.470	**0.176**
(6) Relaxation						1	0.630	0.633
(7) Quality of life							1	0.607
(8) Emotional								1

Values in bold are below or above the recommended criteria (<0.30 to >0.85). 1 = Intrusiveness; 2 = Sense of control; 3 = Cognition; 4 = Sleep; 5 = Auditory; 6 = Relaxation; 7 = Quality of life; 8 = Emotional.

**Table 7C:** ESEM-CFA model: Correlations between factors. Correlations between factors.

Factor	1	2	3	4	5	6	7	8
(1) Intrusiveness	1	0.795	0.691	0.497	0.421	0.6	0.573	0.565
(2) Sense of Control		1	0.688	0.473	0.334	0.596	0.529	0.564
(3) Cognitive			1	0.558	0.542	0.697	0.699	0.621
(4) Sleep				1	**0.268**	0.586	0.48	0.480
(5) Auditory					1	0.399	0.544	**0.292**
(6) Relaxation						1	0.727	0.691
(7) Quality of life							1	0.734
(8) Emotional								1

Values in bold are below or above the recommended criteria (<0.30 to >0.85). 1 = Intrusiveness; 2 = Sense of control; 3 = Cognition; 4 = Sleep; 5 = Auditory; 6 = Relaxation; 7 = Quality of life; 8 = Emotional.

Table 8 shows the results of the goodness of fit test (GOF). Values of root mean square error of approximation (RMSEA) for the three models are below 0.08, indicating good fitting (MacCallum et al., 1996). Despite RMSEA values should normally be below 0.05, the limit of 0.08 is reasonable when the standardized root mean square residual (SRMR) is lower than 0.06 (Hu and Bentler, 1999), which was true for the three models. However, the models ESEM and ESEM-CFA showed better values of GOF. Although AIC and BIC values were smaller for the ESEM-CFA model, these parameters did not differ to a great extent between the three models.

**Table 8:** Goodness of fit (GOF) statistics for the models ICM-CFA, ESEM, and ESEM-CFA.

	ICM-CFA	ESEM	ESEM-CFA
AIC	38197	37951	37913
BIC	38602	38824	38373
RMSEA (90%CI)	0.071 (0.065–0.077)	0.046 (0.036–0.055)	0.044 (0.036–0.051)
SRMR	0.047	0.011	0.027
CFI	0.946	0.988	0.980
TLI	0.943	0.972	0.975

AIC = Akaike information criterion; BIC = Bayesian information criterion; RMSEA = Root Mean Square Error of Approximation; SRMR = Standardised Root Mean Square Residual; CFI = Comparative Fit Index; TLI = Tucker-Lewis Index.

## 4 Discussion

The aim of the present study was to assess the psychometric properties of the Dutch translation of the Tinnitus Functional Index. The original English TFI was translated into Dutch and tested on a population of 377 tinnitus patients. Reliability of the questionnaire was tested by means of internal consistency, and construct validity was estimated through convergent and divergent correlations with the THI and 3 psychological functioning questionnaires. A factor analysis was performed to confirm the 8-factor structure of the TFI by using 3 different models. Overall, the Dutch version of the TFI has shown good qualities with respect to the internal consistency and convergent validity, comparable to the values of the original TFI but also to those obtained for the validation of the Dutch version of the THI (Brussee, 2003).

In line with the study of Meikle (Meikle et al., 2012) and previous validations of the TFI, the Auditory subscale showed the lowest correlation values with the rest of the factors (Wrzosek et al., 2016; Fackrell et al., 2018). A possible explanation of this effect is the comorbidity between tinnitus and hearing loss and the challenge of disentangling the two of them, which is the rationale for the creation of the Tinnitus and Hearing Survey (THS) (Henry et al., 2014). However, the THS addresses tinnitus, hearing and sound tolerance with 4 items per factor. Therefore, the THS takes into account these covariates but is less responsive for assessing tinnitus impact separately. The authors of the original version of the TFI suggested studying the impact of removing the Auditory factor from the questionnaire. This analysis was carried out later by Fackrell et al. (2018), who tested a modified TFI-22 version, which performed better in their UK clinical population. Nevertheless, the authors suggested not removing the Auditory factor from the TFI but using a different scoring system. Taking this into account, we consider that the Auditory factor of the Dutch TFI provides complementary information due to the association between tinnitus and hearing loss and, therefore, it is a useful supplement to the questionnaire. Further studies could investigate the impact of a modified scoring system that increases the Auditory correlation values with the rest of the factors without undermining the TFI performance.

It is noteworthy how the TFI scores were interpreted in the original study of Meikle: mild (scores below 25), moderate (scores between 25 and 50) and severe (scores above 50) problem. As it has been pointed out by Gos et al. (2021), the averaged TFI score is often close to the severe limit, and with a quite high dispersion. This applies in particular to our data (M = 47.93; SD = 20.41), which raises the question of whether the original cut-off for diagnosing severe tinnitus is too low. The study of Gos et al. (2021) suggested that this boundary should be set at 65 points, in order to limit the most severe rating to a smaller sample. In line with Gos et al. findings, 37 % of the patients in our study obtained total score above 50. The study by Fackrell et al. (2016) included both clinical and non-clinical populations, obtaining a lower proportion of participants with global TFI scores above 50 (30 %). The difference in severe cases might be explained by the tinnitus symptoms of a patient population who seek medical help, compared to a general population who might report milder tinnitus on average. In our dataset, a proportion of 17.2 % of patients scored above 65, which is a rather small group and might not represent the distress reported by the patients who visited our clinic. Due to the similarities between the global scores of the THI and the TFI, a potential solution to this problem is to increase the number of categories as in the THI, instead of raising the limit of the group with severe tinnitus.

Construct validity of the Dutch TFI showed strong correlations with the THI for almost all factors. One of the exceptions was the convergent validity between the TFI-factor Intrusive and the THI-factor Emotional. Previous studies highlighted the importance of evaluating tinnitus intrusiveness for studying treatment outcomes (Hoare et al., 2011; Hall et al., 2018). However, tinnitus intrusiveness seems to be a complex construct that can be interpreted in different ways, as it can be deduced from comparing different tinnitus questionnaires (Jacquemin et al., 2019). In the case of the Dutch TFI, the three items belonging to this factor are focused on annoyance, awareness and loudness of the tinnitus percept. Two of these items (awareness and loudness) do not necessarily correlate with the items included in the Emotional factor of the THI, which mostly covers anxiety, depression and psychological impact. The low correlation obtained for this particular comparison between the two questionnaires might be due to this effect, since only one of the items evaluating intrusiveness is clearly connected to the THI-Emotional. A similar effect occurs when comparing the TFI-Auditory to the THI-Functional, for which a weak correlation was obtained as well. The Functional factor covers aspects such as concentration, sleep, intrusiveness and fatigue. Only 2 out of 11 items of this THI factor are surely related to the TFI-Auditory, and these are “Does the loudness of your tinnitus make it difficult for you to hear people?” and “Does your tinnitus interfere with your ability to enjoy your social activities (such as going out to dinner, to the movies)?“. The wide-ranging design of the THI-Functional is presumably the reason for the low convergent validity obtained. Moreover, a strong correlation is expected when comparing two subscales with the same name from different questionnaires, however, they might measure different underlying aspects (Jacquemin et al., 2019). Nevertheless, it should be noted that previous translations of the THI have shown that the subscales are unreliable, and a THI-total scale might be a valid measure of general tinnitus related distress (Zachariae et al., 2000). Further validations of the TFI might benefit most by analyzing construct validities of the global scores.

One aspect of our study that should be considered is the confirmation of the 8-factor structure by means of 3 different models of factor analysis. Most of the available TFI translations used a CFA model based on independent clusters (ICM). This method assumes no crossloadings between factors which leads to poor fit and overestimated factor correlations (Marsh et al., 2009). In addition to this model, the 8-factor structure of the Dutch TFI was confirmed by 2 more models (ESEM and ESEM-CFA) that take into account possible crossloadings between factors and, consequently, further ensuring the fit. This overestimation can be seen when comparing Tables 7B and 8A: all correlations are higher in the first table. We think that the models ESEM and ESEM-CFA are more adequate for a factor analysis in a study like this one, given the complexity and the subjective nature of a tinnitus questionnaire.

Another aspect worth to note with regard to previous TFI translations, is that the Dutch TFI was validated through a broad and diverse clinical population of 377 patients whose characteristics corroborate the values of reliability and construct validity that have been obtained in this study. Both sample size and techniques of factor analysis used in this study make the validation process more robust. It should be noted that the Dutch language used in Rabau et al. (2014) refers to Flemish, which is mostly spoken in Belgium. One of the main motivations of this study was to obtain a new Dutch version that could be fully understood by a clinical population in The Netherlands.

Some items of the models ESEM and ESEM-CFA loaded on to their designated factor but also on to others, resulting in the so-called crossloadings. Although the significance of a factor loading depends on the sample size (Stevens 2012), it's common practice in exploratory factor analysis to ignore loadings below 0.3 (Field et al., 2012). Using the recommendation of Guadagnoli and Velicer (1988), only scores greater than 0.4 are considered stable. In our study, none of the crossloading scores in any of the models exceeded this threshold, resulting in only stable items with loadings on to their designated factor. Despite the crossloadings of both models can be ignored, ESEM-CFA showed better correlations between factors and better GOF values when compared to ESEM. Therefore, we suggest that the ESEM-CFA is the most optimal model out of the three.

Although the Dutch TFI showed a good reliability as a screening tool, responsiveness to treatment for different follow-up groups was not evaluated in this study. The main goals of the original TFI were evaluating both the impact of tinnitus and the treatment-related effects on the patients. Further analyses should focus on evaluating treatment efficacy by measuring the changes before and after treatment for the total score and for each subscale.

Overall, the results of this study show that most of the subscales of the Dutch version of the TFI have a good internal consistency. The reliability scores are considered good for use as a diagnostic instrument as well as a screening instrument (Nunnally, 1994). Furthermore, these results are comparable with the reliability scores of the original TFI (Meikle et al., 2012). Only the subscale “sense of control” showed a low internal consistency, which indicates that its use for screening should be done carefully, although the scale is acceptable for using it as a research instrument.

## Declarations

### Author contribution statement

Jose L. Santacruz: Analyzed and interpreted the data; Contributed reagents, materials, analysis tools or data; Wrote the paper.

Rosemarie Arnold: Conceived and designed the experiments; Performed the experiments; Analyzed and interpreted the data; Contributed reagents, materials, analysis tools or data; Wrote the paper.

Jolanda Tuinstra: Performed the experiments; Analyzed and interpreted the data.

Roy E. Stewart: Analyzed and interpreted the data; Wrote the paper.

Pim van Dijk: Conceived and designed the experiments; Analyzed and interpreted the data; Contributed reagents, materials, analysis tools or data; Wrote the paper.

### Funding statement

This work was supported by the 10.13039/100010661European Union’s Horizon 2020 research and innovation programme under the Marie Sklodowska-Curie grant agreement number 722046.

### Data availability statement

The authors do not have permission to share data.

### Declaration of interests statement

The authors declare no conflict of interest.

### Additional information

No additional information is available for this paper.
